# Dissatisfaction Risk Factors of Patients after Laminectomy for Thoracic Ossification of Ligamentum Flavum: A Retrospective Cohort Study of Different Follow-Up Periods

**DOI:** 10.1155/2021/3971396

**Published:** 2021-12-20

**Authors:** Zhiwei Wang, Sidong Yang, Xianda Gao, Zheng Wang, Wenyuan Ding, Dalong Yang

**Affiliations:** ^1^Department of Spine Surgery, The Third Hospital of Hebei Medical University, 139 Ziqiang Road, Shijiazhuang 050051, China; ^2^Hebei Provincial Key Laboratory of Orthopaedic Biomechanics, 139 Ziqiang Road, Shijiazhuang 050051, China

## Abstract

**Objectives:**

To explore the influencing factors of satisfaction with postoperative treatment in patients diagnosed with thoracic ossification of the ligamentum flavum during different follow-up periods.

**Methods:**

This was a retrospective study of 57 patients who were diagnosed with thoracic ossification of ligamentum flavum (TOLF) and treated with laminectomy in the Spine Surgery Department of the Third Hospital of Hebei Medical University from January 2010 to January 2017. The Patient Satisfaction Index (PSI) was collected at discharge and at 6-month, 1-year, and the last follow-up. According to the evaluation results, the patients could be divided into a satisfied group and a dissatisfied group. The patient's Japanese Orthopaedic Association (JOA) score improvement rate was evaluated at the last follow-up. Possible influencing factors of the two groups of patients were compared and the related influencing factors of satisfaction with postoperative treatment in patients during different follow-up periods were summarized.

**Results:**

At the time of discharge, the dissatisfied and satisfied groups had significant differences in variables of diabetes mellitus, duration of preoperative symptoms, urination disorder, intramedullary signal change on MRI, dural ossification, residual rate of cross-sectional spinal canal area on CT, shape on the sagittal MRI, hospital stay, hospitalization expenses, postoperative pain in LE VAS, delayed wound healing, postoperative depression, and intercostal pain (*P* < 0.05). There were also significant differences in urination disorder, postoperative pain according to the LE VAS, JOA score, and postoperative depression during the 6-month follow-up (*P* < 0.05). There were no significant differences in other variables between the two groups (*P* > 0.05). One year after the operation, there were significant differences between the dissatisfied group and the satisfied group in urination disorder, JOA score, and symptom recurrence (*P* < 0.05). There were also significant differences in the JOA score and symptom recurrence at the final follow-up (*P* < 0.05). For further analysis, the duration of preoperative symptoms in the satisfied group was less than 24 months and the duration of preoperative symptoms in the dissatisfied group was more than 24 months. The JOA scores of patients in the satisfied group and the dissatisfied group increased gradually with the improvement of neurological function in different follow-up periods, but, at the last follow-up, the JOA scores of patients in the satisfied group were significantly higher than those in the dissatisfied group.

**Conclusions:**

In conclusion, for thoracic ossification of ligamentum flavum patients who received laminectomy, dissatisfaction with the early and medium-term postoperative results may be related to diabetes, the duration of preoperative symptoms, hospitalization expenses, delayed wound healing, intercostal pain, and urination disorder, and dissatisfaction with the long-term postoperative results might be related to the low JOA score improvement rate and symptom recurrence.

## 1. Introduction 

**Figure 1:**
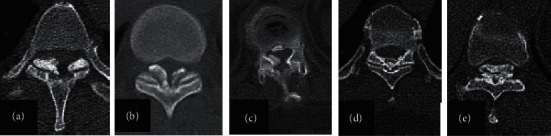
Sato's classification of TOLF. A-E Sato's classification: (a) lateral type, (b) extended type, (c) enlarged type, (d) fused type, and (e) tuberous type.

**Figure 2:**
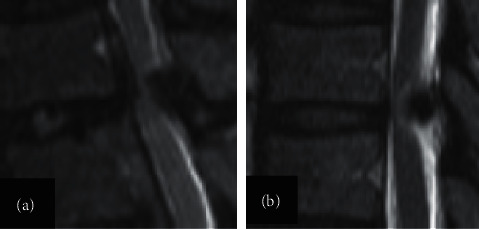
The shape on the sagittal MRI of TOLF. Sagittal T2-weighted MR images demonstrating morphological classifications of TOLF: (a) beak type and (b) round type.

Thoracic ossification of the ligamentum flavum (TOLF) is a pathological heterotopic ossification that mainly occurs in the lower thoracic spine, namely, T10∼11, T9∼10, and T11∼12 [1–5]. According to Sato's classification [6], TOLF can be divided into five types: lateral type, extended type, enlarged type, fused type, and tuberous type (Figure 1). According to the shape on the sagittal MRI [7], it was divided into a beak or round type (Figure 2). Once TOLF is diagnosed, surgical decompression should be performed as soon as possible [8]. Traditional open surgery includes posterior laminectomy, laminectomy with internal fixation, and laminoplasty [9, 10].

Previous studies have shown that the postoperative effect of TOLF is sometimes dissatisfactory due to multiple risk factors [10]. There were differences in patients' attitudes towards diseases between the satisfied and the dissatisfied groups. Patients in the satisfied group cooperated with medical institutions with a positive attitude and provided important medical information to obtain satisfactory medical services. Because of the decrease in some pre- and postoperative risk factors, the treatment effect was better. In contrast, dissatisfied patients were often unwilling to communicate their illness with medical workers, did not actively seek treatment, or refused to follow the prescribed course of treatment, resulting in a poor treatment effect. As patients' dissatisfaction with the postoperative effect will affect the evaluation of the therapeutic effect, the analysis of the effect factors of patients' postoperative satisfaction can better evaluate the postoperative effect, provide correct and effective treatment for the patients, and make TOLF patients satisfied with the surgical treatment.

## 2. Materials and Methods

### 2.1. General Information

The clinical data of 57 patients (35 males and 22 females) with TOLF diagnosed by spinal surgery in The Third Hospital of Hebei Medical University who underwent laminectomy from January 2010 to January 2017 were reviewed. Inclusion criteria: 1. patients who were diagnosed with TOLF and treated with laminectomy; 2. follow-up time of two years or more. Exclusion criteria: 1. patients with thoracic disc herniation; 2. patients with cervical and lumbar spine diseases; 3. patients with trauma, inflammation, infection, or tumours affecting the spine. Of the 57 patients included in the study, 40 underwent laminectomy alone, and 17 underwent laminectomy with instrumentation. A total of 57 patients (35 males and 22 females) were included in the study and underwent laminectomy (40) and laminectomy with internal fixation (17). This study was approved by the ethics committee of our hospital, and informed consent was obtained from all patients included in the study.

### 2.2. Study Variables

Age, sex, BMI, smoking history, drinking history, heart disease, hypertension, and diabetes mellitus were collected before surgery. Surgical study variables should include operation time, duration of preoperative symptoms, OLF segment, number of ossified segments, wound length, urination disorder, intramedullary signal change on MRI, dural ossification, residual rate of cross-sectional spinal canal area on CT, Sato's classification, shape on the sagittal MRI, blood loss, hospitalization expenses, pre- and postoperative JOA score, postoperative pain in LE VAS, mode of operation, and hospitalization days. Complications were collected at different follow-up periods. We chose a 2-year follow-up interval because we wanted to evaluate patient satisfaction within the time when the desired postoperative outcome was expected.

### 2.3. Operation Method

The operations of all patients included in the study were performed by the same surgeon. The relevant tissue structure was exposed (for patients who underwent laminectomy with internal fixation, the pedicle was screwed first); the spinous process, part of the articular process, and lamina were removed and then a drill was used to grind most of the lateral lamina and related parts. The tissue was ossified; the field of vision was fully exposed, and then a nerve stripper was used to explore whether the OLF adhered to the dura mater. If there was no adhesion, the OLF was held with laminar forceps and decompressed thoroughly. After the surgical decompression was completed (patients who underwent laminectomy with internal fixation installed the precurved connecting rod on the pedicle screw and tightened it to lock), the surgical field was flushed, the drainage tube indwelled and sutured layer by layer. The operation was thus completed.

### 2.4. Satisfaction Evaluation

The relevant variables of each patient were counted by different staff rather than surgeons to avoid bias. A preoperative demographic questionnaire was provided to patients at the time of admission. In different follow-up periods, patients' satisfaction with postoperative efficacy was different. We followed up with patients through outpatient clinics or telephone and evaluated the Patient Satisfaction Index (PSI) [11]. Patients who chose 1 or 2 were included in the satisfaction group, and patients who chose 3 or 4 were included in the dissatisfaction group (Table 1). Evaluation of neurological function was assessed with the Japanese Orthopaedic Association (JOA) score. Evaluation of thoracic spinal cord recovery function was performed with the modified JOA score [12, 13] (Table 2). Evaluation of the neurological function improvement rate during follow-up: JOA score improvement rate = (JOA score at follow-up preoperative JOA score)/(11-preoperative JOA score) × 100%. Efficacy was defined according to the improvement rate of the JOA score [14]: excellent (≥75%), good (50%–74%), general (25%–49%), and poor (<25%). In addition, we believe that the cognitive level of TOLF was also related to family income, region (rural or urban), educational level, and occupation. An in-depth understanding of these background differences was helpful to further analyze the influencing factors of patients' satisfaction with postoperative efficacy.

### 2.5. Statistical Analysis

All statistical analyses were carried out by SPSS software version 22.0 (IBM, Armonk, NY, USA), and the test level was *α* = 0.05. The measurement data between the two groups were compared by independent sample *t*-tests or nonparametric tests according to whether they were in line with a normal distribution and homogeneity of variance. Analysis of counting data was carried out by the chi-square test. Significance was accepted at *p* < 0.05.

## 3. Results

Fifty-seven patients with TOLF were treated with laminectomy (40 patients with open laminectomy and 17 patients with open laminectomy and internal fixation). All patients were followed up with for 2 years. The average JOA score recovery rate was 54.78 ± 17.62% at the last follow-up.

Twenty-seven patients (47.4%) in the dissatisfied group were compared with those in the satisfied group at discharge. Diabetes mellitus, duration of preoperative symptoms, urination disorder, intramedullary signal change on MRI, dural ossification, residual rate of cross-sectional spinal canal area on CT, shape on the sagittal MRI, hospital stay, hospitalization expenses, postoperative pain in LE VAS, delayed wound healing, postoperative depression, and intercostal pain had significant differences (*P* < 0.05). There were no significant differences between the two groups in other variables (*P* > 0.05) (Tables 3, 4). For further analysis, the duration of preoperative symptoms in the satisfied group was less than 24 months and that in the dissatisfied group was more than 24 months. In the dissatisfied group, the incidence of cerebrospinal fluid leakage caused by dural tears during the operation (33.33%) was higher than that of patients without dural ossification (13.33%). The incidence of delayed wound healing in dissatisfied patients with diabetes (75.00%) was higher than that in patients without diabetes (26.67%). In addition, the patients in the dissatisfied group had long hospitalization days, high hospitalization costs, and a high incidence of postoperative depression. These may be the influencing factors of patients' early dissatisfaction.

Twenty-two patients (38.6%) in the dissatisfied group in terms of urination disorder, postoperative pain in LE VAS, JOA score, and postoperative depression were significantly different from those in the satisfied group during the 6-month follow-up (*P* < 0.05). There were no significant differences between the two groups in other variables (*P* > 0.05) (Table 5).

**Figure 3:**
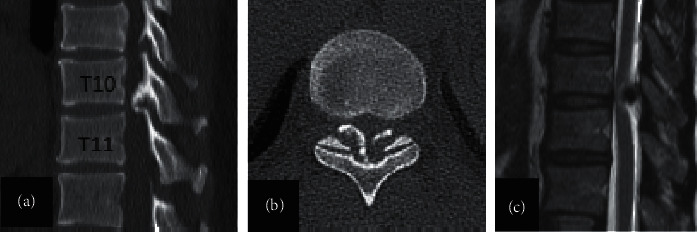
A 62-year-old male patient of the dissatisfied group. A 62-year-old male patient (a, b, and c) of the dissatisfied group Ossification of the ligamentum flavum at the T10–11 intervertebral disc level. The main preoperative symptoms of the patients were numbness, weakness of both lower limbs, and difficulty in walking with urination disorder for more than 2 years. Physical examination showed a sense of banding in the chest and abdomen, knee hyperreflexia, and Babinski sign (+). He was performed with laminectomy without related complications. At 6-month and 1-year follow-up, the symptoms of significantly limited physical activity were relieved. However, the improvement of urination disorder was not obvious, which had been troubling the patients after operation and seriously affected the quality of life of the patients. At the last follow-up, her JOA scores improved from 3 before operation to 7 after operation.

One year after the operation, 14 patients (24.6%) in the dissatisfied group in terms of urination disorder, JOA score, and symptom recurrence were significantly different from those in the satisfied group (*P* < 0.05). There were no significant differences between the two groups in other variables (*P* > 0.05) (Table 6). At the last follow-up, eight patients (14.1%) in the dissatisfied group in terms of JOA score and symptom recurrence were significantly different from those in the satisfied group (*P* < 0.05) (Table 7). For further analysis, the JOA scores of patients in the satisfied group and the dissatisfied group increased gradually with the improvement of neurological function in different follow-up periods, but, at the last follow-up, the JOA scores of patients in the satisfied group were significantly higher than those in the dissatisfied group (Figure 3).

## 4. Discussion

TOLF has become one of the main causes of chronic thoracic spinal cord injury [15, 16]. TOLF often has a slow onset [8], and its incidence is the highest in people aged 50 to 59 and increases with age [17]. Early diagnosis is more difficult [18]. The incidence in the population and pathogenesis of TOLF are not clear, and they are mainly concentrated in Asia, with more reports in Japan [19–21]. Wang et al. [22] analyzed 142 patients with thoracic ligamentum flavum ossification and found that TOLF was related to systemic ossification diseases, changes in spinal load, and ageing. Conservative treatment of TOLF is ineffective. Once diagnosed, surgical treatment should be performed as soon as possible. In the study of Kang et al. [10], 20 (39%) of 51 patients with TOLF undergoing surgery were dissatisfied with the effect of surgery. This study only proposed an overall degree of dissatisfaction and did not specifically describe the relevant influencing factors. We performed a more detailed follow-up of 57 TOLF patients who received laminectomy to explore the influencing factors of postoperative efficacy satisfaction, which has more advantages. Our results showed that dissatisfaction with the early and medium-term postoperative results might be mainly related to diabetes, the duration of preoperative symptoms, hospitalization expenses, delayed wound healing, intercostal pain, and urination disorder, and dissatisfaction with the long-term postoperative results might be mainly related to the low JOA score improvement rate and symptom recurrence.

Our study found that, at discharge, the hospitalization costs of the dissatisfied group were higher than those of the satisfied group, which might be because the patients in the dissatisfied group had poor postoperative neurological function recovery and needed more treatment, such as drugs, to promote neurological recovery and professional rehabilitation training. All these factors would increase the cost of hospitalization, especially for patients with low family income. The high cost of hospitalization often costs their income for one year or even several years, and the huge financial burden often makes them feel dissatisfied with the operation. With the improvement of postoperative neurological function, patients need to bear increasingly lower rehabilitation costs. At the 6-month follow-up, there was no significant difference in hospitalization costs between the satisfied and the dissatisfied groups. In addition, patients with different cultural backgrounds and different regions (rural or urban) might have different perceptions of their satisfaction with the postoperative efficacy. For patients, especially, with low education from rural areas, their cognitive level of TOLF was limited. In their cognitive thinking, the expected effect after the operation was higher; that is, they could live like normal people after ossification of ligamentum flavum. Once the operation did not live up to their expectations, they became very disappointed. Patients who had a certain cognitive level of TOLF often had a certain understanding of the disease through various channels. Therefore, they were psychologically prepared for the postoperative effect. Even if the operation failed to meet their expectations, they would cooperate with the rehabilitation with a positive attitude.

We found that diabetes mellitus, duration of preoperative symptoms, urination disorder, intramedullary signal change on MRI, dural ossification, residual rate of cross-sectional spinal canal area on CT, and shape on sagittal MRI were important factors affecting patients' early and middle satisfaction.

Diabetes is a type of metabolic disease with multiple causes that affects all organs of the body, including bone, joint, and cartilage [23]. The pathogenesis of ossification of the ligamentum flavum caused by diabetes remains unclear. Moon et al. [24] suggested that BMP-2 could induce the expression of normal human ligamentum flavum cells and osteogenic genes and the formation of calcium nodules. Yokosuka et al. [25] found that hyperglycaemia caused by diabetes could induce the production of BMP-2. Braddock et al. [26] found that the incidence of TOLF in diabetic mice was higher than that in nondiabetic mice. In previous TOLF studies, there were few reports on whether diabetes affects postoperative satisfaction. We found that the number of patients with diabetes in the dissatisfied group (12 cases) was significantly higher than that in the satisfied group (five cases). The incidence of delayed wound healing in the dissatisfied group of patients with diabetes mellitus (75.00%) was higher than that in patients without diabetes mellitus (26.67%). Patients with diabetes might experience vascular calcification because their blood sugar was high for a long time. Even if the ossified ligamentum flavum had been removed and the compression had been relieved, the blood circulation around the spinal cord remained poor, resulting in poor recovery of postoperative neurological function, which might cause dissatisfaction of the patients.

Previous studies have suggested that the duration of preoperative symptoms is an important postoperative predictor [16, 27]. He et al. [28] found that the duration of preoperative symptoms and JOA score were also important predictors for evaluating postoperative efficacy. Our study found that patients with a duration of preoperative symptoms >24 months (26.37 ± 12.46) had lower preoperative JOA scores than patients with a duration of preoperative symptoms <24 months (18.93 ± 10.12). The results show that the timely surgical treatment of TOLF patients whose preoperative duration of symptoms is <24 months can obtain satisfactory postoperative effects.

The residual rate of the cross-sectional spinal canal area on CT can indicate the degree of invasion of the ossified ligamentum flavum to the spinal canal. Ossification of the ligamentum flavum leads to spinal canal stenosis and spinal cord compression, which seriously impairs the blood supply of the spinal cord [29]. After the removal of ossification, ischaemia-reperfusion injury may occur, resulting in the deterioration of postoperative neurological function and poor surgical effect. Sanghvi et al. [30], through a study of 25 patients with TOLF, found that the residual rate of the spinal canal area was positively correlated with the postoperative effect. In our study, we found that the residual rate of the cross-sectional spinal canal area on CT was closely related to patient satisfaction with the postoperative effect. In the dissatisfied group, 23 patients had a residual rate of cross-sectional spinal canal area on CT ≤ 60%.

The changes in the intramedullary signal on MRI showed intramedullary high signal intensity on T2WI. The pathological manifestations were oedema of grey or white mater, loss of nerve cells, Wallerian degeneration, glial hyperplasia, and demyelination [2, 31]. Kuh et al. [7] considered that the intramedullary signal change caused by beak lesions was a factor of poor prognosis by studying the shape on sagittal MRI (beak or round type). In our study, 17 of the 25 patients with intramedullary signal changes in the dissatisfied group were caused by beak lesions, which might be severe spinal cord injury caused by ossification of the ligamentum flavum. However, during follow-up, we were unable to observe whether the intramedullary high signal disappeared after the operation on MRI, which might indicate whether the intramedullary signal changes were reversible after resection of the ossified ligamentum flavum. In addition, He et al. [28] showed that patients with urination disorder had poor postoperative prognosis. A multicentre study by Ando et al. [32] found that dural ossification was associated with surgical prognosis. We found that TOLF patients with urination disorder and dural ossification were dissatisfied with the postoperative effect.

The low improvement rate of the JOA score is one of the adverse factors that cause dissatisfaction of patients with long-term postoperative efficacy. The improvement rate of the JOA score reflects the patient's postoperative neurological recovery. A low improvement rate in the JOA score might be due to severe compression of the spinal cord caused by ossification of the ligamentum flavum before the operation and poor recovery of neurological function after the operation. This makes the patients feel that preoperative symptom relief was not obvious; for example, urination disorders still existed, which seriously affected their quality of life. For some patients, it even increased the risk of reoperation, which brought great physical and mental harm to the patients and even made the patients think that the operation was ineffective and feel very desperate, making it difficult for them to accept the outcome. Kang et al. [10] found that the average preoperative JOA score of patients was 5.5 points, and the last follow-up was 7.4 points. The results of our study were consistent; that is, the JOA score at the last follow-up was higher than that before the operation. Furthermore, we also found that, at the last follow-up, the average JOA score of the dissatisfied group was significantly lower than that of the satisfied group. The JOA score had no significant effect on satisfaction in the early and middle stages because patients were hopeful about the postoperative effect during this period. However, as symptom relief was not obvious after one year, the effect of a lower JOA score improvement rate on quality of life gradually emerged. This greatly increased patients' dissatisfaction with the postoperative effect.

Symptom recurrence was another adverse factor for long-term dissatisfaction of patients. During the operation, we sent the excised ossified ligamentum flavum to pathological examination in a timely manner. The pathological results showed that the elastic fibres of the ligamentum flavum decreased and were arranged in a disorderly manner, and the partially ossified collagen fibres and chondrocytes proliferated actively. We considered TOLF as a chronic progressive disease. Further analysis suggested that decompression might only remove the ossified segments, but the progress of the disease itself had not stopped. At the last follow-up, 10 cases of recurrence were observed in 57 patients in this group. We found that the patients with recurrent symptoms in the dissatisfied group had greater mood swings and were anxious about resection of the ossified ligamentum flavum and thought that the operation was a failure. They had a strong resistance to the operation and were unwilling to undergo a second operation. Symptom recurrence seriously affected patient satisfaction with postoperative efficacy. Patients with symptom recurrence in the satisfied group, because they had a certain understanding of the progress of TOLF before the operation and had a full understanding of the effect of neurological recovery after the operation, even if symptom recurrence occurred, would cooperate with medical institutions in a positive attitude to seek better solutions.

This study has several limitations. First, the sample size of the study we included was small, and the follow-up time was short, so we look forward to a study with a large sample size and long-term follow-up to further confirm our conclusions. Second, patient satisfaction is the patient's self-perception and evaluation of clinical diagnosis and treatment, which is affected by multiple factors, such as the postoperative efficacy, whether the medical cost is reasonable, and whether the pain has been resolved through medical treatment. Different patients have different subjective feelings about the satisfaction of the postoperative effect; that is, there may be a selection deviation in evaluating the satisfaction of patients. Furthermore, the PSI is simple and easy to use to evaluate postoperative efficacy satisfaction, but it may not be specific enough. We also believe that changes in the ossified ligamentum flavum and intramedullary signals play an important role in the recovery of long-term neurological function. However, due to the retrospective nature of the study, there was no routine imaging review of postoperative patients. We hope to elaborate on this point in future studies.

## 5. Conclusion

Overall, for TOLF patients who received laminectomy, dissatisfaction with the early and medium-term postoperative results might be related to diabetes, the duration of preoperative symptoms, hospitalization expenses, delayed wound healing, intercostal pain, and urination disorder. Dissatisfaction with the long-term postoperative results may be related to the low JOA score improvement rate and symptom recurrence.

## Figures and Tables

**Table 1:** Patient Satisfaction Index (PSI).

PSI	Patient responses
1	Surgery met my expectations
2	Surgery improved my condition enough so that I would go through it again for the same outcome
3	Surgery helped me but I would not go through it again for the same outcome
4	I am the same or worse compared to before surgery

**Table 2:** Modified Japanese Orthopaedic Association (JOA) scoring system for the assessment of thoracic myelopathy (total score 11 points).

Functional score	
Motor function: lower limb	
0	Unable to walk.
1	Support was needed to walk on flat ground.
2	Need a cane or aid on flat ground.
3	Walking on flat ground or upstairs did not require support, but the lower limbs were not flexible.
4	Normal.

Sensory function: lower limb	
0	Obvious sensory impairment.
1	Mild sensory impairment or numbness.
2	Normal.

Sensory function: trunk	
0	Obvious sensory impairment.
1	Mild sensory impairment or numbness.
2	Normal.

Bladder function	
0	Uroschesis.
1	Highly dysuria, laborious, irretention, or incontinence.
2	Mild dysuria, frequent urination, hesitation in urination.
3	Normal.

**Table 3:** The main demographic variables in the satisfied and dissatisfied patients at discharge.

Characteristics	Satisfaction (*n* = 30, 52.6%)	Dissatisfaction (*n* = 27, 47.4%)	*P* Value
Age (years)	58.57 ± 8.39	60.18 ± 6.97	0.434
Sex (male/female)	18/12	17/10	0.819
BMI (kg/m^2^) (≤27/>27)	15/15	13/14	0.889
Smoking (yes/no)	10/20	11/16	0.563
Drinking (yes/no)	7/23	5/22	0.656
Heart disease (yes/no)	6/24	8/19	0.399
Hypertension (yes/no)	8/22	9/18	0.583
Diabetes (yes/no)	5/25	12/15	0.022^*∗*^

BMI  Body Mass Index, and ^*∗*^the difference possessing statistical significance ^*∗*^*P* < 0.05.

**Table 4:** The related risk factors of satisfied and dissatisfied patients at discharge.

Characteristics	Satisfaction (*n* = 30, 52.6%)	Dissatisfaction (*n* = 27, 47.4%)	*P* Value
Preoperative duration of symptoms (months)	18.93 ± 10.12	26.37 ± 12.46	0.016^*∗*^

OLF segment			0.133
Upper thoracic (T1–4)	2	6	
Middle thoracic (T5–8)	9	10	
Lower thoracic (T9–12)	19	11	

Number of ossified segments			0.195
1	21	13	
2	7	9	
>2	2	5	

Urination disorder			0.005^*∗*^
Yes	2	10	
No	28	17	

Intramedullary signal change on MRI			0.009^*∗*^
Yes	19	25	
No	11	2	

Dural ossification			0.001^*∗*^
Yes	2	12	
No	28	15	

Residual rate of cross-sectional spinal canal area on CT			0.035^*∗*^
≤60%	18	23	
＞60%	12	4	

Sato′s classification			0.241
Lateral type	7	3	
Extended type	13	7	
Enlarged type	5	7	
Fused type	2	5	
Tuberous type	3	5	

Shape on the sagittal MRI			0.031^*∗*^
Beak	27	18	
Round	3	9	

Operation time (min)			0.217
≤250	16	10	
＞250	14	17	
Blood loss (ml)	676.67 ± 335.21	825.93 ± 405.34	0.134
Hospital stay (days)	10.47 ± 1.93	13.07 ± 2.49	<0.001^∗∗^
Wound length (cm)	16.6 ± 1.5	17.2 ± 2.1	0.264
Hospitalization expenses (thousand RMB)	49.1 ± 8.4	54.3 ± 10.2	0.041^*∗*^
Pre-mJOA	5.37 ± 1.45	4.74 ± 1.51	0.116
Post-mJOA	6.23 ± 1.48	5.81 ± 1.52	0.297
Preoperative pain in LE VAS	4.27 ± 0.69	4.41 ± 0.84	0.492
Postoperative pain in LE VAS	3.03 ± 1.09	4.14 ± 0.95	<0.001^∗∗^

Mode of operation			0.542
Laminectomy alone	20	20	
Laminectomy with instrumentation	10	7	
Complications			

Wound infected			0.144
Yes	5	9	
No	25	18	

Delayed wound healing			0.004^*∗*^
Yes	4	13	
No	26	14	

Leakage of cerebrospinal fluid			0.091
Yes	2	6	
No	28	21	
Neurological deficit	Nil	Nil	

Postoperative depression			0.006^*∗*^
Yes	8	17	
No	22	10	

Intercostal pain			0.026^*∗*^
Yes	9	16	
No	21	11	

Thrombosis of lower extremities			0.587
Yes	3	5	
No	27	22	

Others			0.229
Yes	4	7	
No	26	20	

mJOA  Modified Japanese Orthopaedic Association, VAS  Visual Analog Scale, LE : lower extremity, and ^*∗*^the difference possessing statistical significance ^*∗*^*P* < 0.05,^∗∗^*P* ≤ 0.001.

**Table 5:** The related risk factors of satisfied and dissatisfied patients at the 6-month follow-up.

Characteristics	Satisfaction (*n* = 35, 61.4%)	Dissatisfaction (*n* = 22, 38.6%)	*P* Value
Diabetes			0.147
Yes	8	9	
No	27	13	

Urination disorder			0.024^*∗*^
Yes	2	7	
No	33	15	

Residual rate of cross-sectional spinal canal area on CT			0.134
≤60%	3	5	
>60%	32	17	
Hospitalization expenses (thousand RMB)	3.54 ± 0.81	3.79 ± 0.68	0.246
mJOA scores after 6 months	6.89 ± 1.51	6.04 ± 1.32	0.037^∗^
VAS pain in LE after 6 months	2.03 ± 1.25	3.22 ± 1.19	0.001^*∗*^
Complications			

Delayed wound healing			0.444
Yes	4	5	
No	31	17	

Postoperative depression			0.025^*∗*^
Yes	3	8	
No	32	14	

Intercostal pain			0.057
Yes	2	5	
No	33	17	

Symptom recurrence			0.677
Yes	1	2	
No	34	20	

mJOA  Modified Japanese Orthopaedic Association, VAS  Visual Analog Scale, LE : lower extremity, and ^*∗*^the difference possessing statistical significance ^*∗*^*P* < 0.05.

**Table 6:** The related risk factors of satisfied and dissatisfied patients at 1-year follow-up.

Characteristics	Satisfaction (*n* = 43, 75.4%)	Dissatisfaction (*n* = 14, 24.6%)	*P* Value
Urination disorder			0.042^*∗*^
Yes	2	4	
No	41	10	

mJOA scores after 1 year	7.46 ± 1.24	6.57 ± 1.34	0.026^*∗*^
VAS pain in LE after 1 year	2.02 ± 1.06	2.21 ± 1.25	0.577
Complications			

Postoperative depression			0.095
Yes	3	4	
No	40	10	

Symptom recurrence			0.025^*∗*^
Yes	3	5	
No	40	9	

mJOA  Modified Japanese Orthopaedic Association, VAS  Visual Analog Scale, LE : lower extremity, and ^*∗*^the difference possessing statistical significance ^*∗*^*P* < 0.05

**Table 7:** The related risk factors of satisfied and dissatisfied patients at 2-year follow-up.

Characteristics	Satisfaction (*n* = 49, 85.9%)	Dissatisfaction (*n* = 8, 14.1%)	*P* Value
Urination disorder			0.370
Yes	2	1	
No	47	7	

mJOA scores after 2 years	9.31 ± 0.84	6.87 ± 1.55	<0.001^∗∗^
VAS pain in LE after 2 years	2.02 ± 0.98	2.13 ± 0.84	0.779
Complications			

Postoperative depression			0.263
Yes	1	1	
No	48	7	

Symptom recurrence			<0.001^∗∗^
Yes	4	6	
No	45	2	

mJOA  Modified Japanese Orthopaedic Association, VAS  Visual Analog Scale, LE : lower extremity, and ^*∗*^the difference possessing statistical significance ^*∗*^*P* < 0.05,^∗∗^*P* ≤ 0.001.

## Data Availability

The data used to support the findings of this study are available from the corresponding author upon request.

## Abbreviations

TOLF: Thoracic ossification of ligamentum flavum
PSI: Patient Satisfaction Index
CT: Computed tomography
MRI: Magnetic resonance imaging
JOA: Japanese Orthopaedic Association
VAS: Visual analog scale
LE: Lower extremity
